# Melatonin Signaling Controls the Daily Rhythm in Blood Glucose Levels Independent of Peripheral Clocks

**DOI:** 10.1371/journal.pone.0148214

**Published:** 2016-01-29

**Authors:** Sharon Owino, Susana Contreras-Alcantara, Kenkichi Baba, Gianluca Tosini

**Affiliations:** Neuroscience Institute and Department of Pharmacology and Toxicology, Morehouse School of Medicine, Atlanta, Georgia, United States of America; McGill University, CANADA

## Abstract

Melatonin is rhythmically secreted by both the pineal gland and retina in a circadian fashion, with its peak synthesis occurring during the night. Once synthesized, melatonin exerts its effects by binding to two specific G-protein coupled receptors–melatonin receptor type 1(MT_1_) and melatonin receptor type 2(MT_2_). Recent studies suggest the involvement of MT_1_ and MT_2_ in the regulation of glucose homeostasis; however the ability of melatonin signaling to impart timing cues on glucose metabolism remains poorly understood. Here we report that the removal of MT_1_ or MT_2_ in mice abolishes the daily rhythm in blood glucose levels. Interestingly, removal of melatonin receptors produced small effects on the rhythmic expression patterns of clock genes within skeletal muscle, liver, and adipose tissue. Taken together, our data suggest that the loss of the daily rhythm in blood glucose observed in MT_1_^-/-^ and MT_2_^-/-^ mice does not occur as a consequence of ‘disrupted’ clocks within insulin sensitive tissues. Finally our results highlight a diurnal contribution of melatonin receptor signaling in the daily regulation of blood glucose levels.

## Introduction

Melatonin is synthesized by both the pineal gland and retina, and functions to drive the temporal variation in a vast array of physiological processes from sleep/wake cycles to reproductive physiology. In mammals, both its synthesis and secretion occurs at night, under the control of the circadian clock located within the suprachiasmatic nucleus (SCN) of the hypothalamus [1]. Once synthesized, melatonin exerts its effects by binding two specific G-protein coupled receptors–melatonin receptor type 1(MT_1_) and melatonin receptor type 2(MT_2_). Both MT_1_ and MT_2_ have been shown to activate several signaling pathways, most notably the Gi/cAMP and Gq/Phospholipase C (PLC)/Ca^2+^ pathways [2,3].

Several lines of evidence suggest a role for melatonin receptors in the regulation of glucose metabolism. Indeed, both MT_1_ and MT_2_ are present within the pancreas where they appear to exert a predominantly inhibitory effect on insulin secretion via receptor mediated attenuation of adenylate cyclase and guanylate cyclase [4–7]. In addition, in vitro studies utilizing a glucagon producing α-cell line, demonstrate that melatonin produces a direct stimulatory effect on glucagon secretion via a PLC dependent mechanism [8]. This secretory response was blocked in the presence of melatonin receptor antagonists; thus demonstrating that MT_1_ and MT_2_ within pancreatic islets are coupled to signaling pathways involved in the modulation of both insulin and glucagon secretion.

It has also been postulated that signaling through melatonin receptors is capable of enhancing systemic glucose tolerance via a more direct effect on glucose uptake. In support of this notion, in vitro studies have reported that melatonin administration stimulates glucose uptake in both skeletal muscle and adipose tissue [9,10]. Furthermore a recent study utilizing melatonin receptor knock-out mice, demonstrates that removal of MT_1_ leads to the development of insulin resistance and glucose intolerance [11]. In line with data obtained in rodents, recent human genetic studies link polymorphisms in MT_1_ and MT_2_ to increased risk of developing insulin resistance and diabetes [12–16].

One of the most pronounced rhythmic aspects of physiology within both humans and rodents is the daily regulation of blood glucose levels across the day [17–21]. Studies now confirm the presence of clock genes within many peripheral tissues [22] where they function to regulate important physiological and metabolic outputs [23]. Additional experimental evidence has also demonstrated that neuronal and humoral signals emanating from the SCN synchronize the expression pattern of these genes in peripheral tissues [22,23]. Interestingly, melatonin—via MT_1_ receptor signaling—has been implicated in the regulation of clock gene transcription within many peripheral tissues [24–26].

To date, relatively few studies have characterized the effect of melatonin on the temporal organization of glucose metabolism. Removal of endogenous melatonin levels by pinealectomy alters the daily rhythm in blood glucose levels by increasing night-time glucose concentrations[27]; and MT_1_^-/-^ mice display higher mean blood glucose levels over a 24 hour period[28]. It remains unclear how melatonin exerts its control on the temporal regulation of blood glucose levels and to what extent this regulation is controlled by the ability of melatonin to synchronize clock genes within insulin sensitive tissues. Therefore we hypothesized that mice lacking melatonin receptors will display altered daily rhythms in blood glucose levels as a consequence of ‘disrupted’ clocks within insulin sensitive tissues. Here we report that removal of MT_1_ and MT_2_ in mice abolishes daily rhythms in blood glucose levels independently of its effects on peripheral tissue clocks in skeletal muscle, adipose tissue, and the liver. Furthermore, MT_1_^-/-^ and MT_2_^-/-^ mice exhibit a clear daytime blunting in the amplitude of their blood glucose rhythms- thereby demonstrating that the effects of melatonin receptor deletion are not restricted to the night phase.

## Materials and Methods

### Sample collection and Blood glucose measurements

Melatonin proficient (C3H-f+/+; WT) and melatonin proficient mice lacking MT_1_ (C3H-f+/+; MT_1_^-/-^) or MT_2_ (C3H-f+/+; MT_2_^-/-^) were used in this study [29]. Male mice (3–5 months) maintained in a 12 hour Light: Dark (LD) cycle (lights on at 7am [denoted ZT0] and lights off at 7pm [ZT12]), food and water were available ad libitum. For tissue collection, mice were anesthetized by isoflurane, and then killed by cervical dislocation every 3 hours over a span of 24 hours. Skeletal muscle (quadricep), liver, and white adipose tissue (epididymal fat) were collected with sterile forceps, immediately frozen on dry ice, and stored at -80°C until use. All experimental procedures were performed in accordance with the NIH Guide on Care and Use of Laboratory Animals and were approved by the Morehouse School of Medicine Animal Care and Use Committee.

In a separate time course study blood glucose levels were measured every 3 hours over a span of 24 hours. Separate animals were used for each time point to avoid inducing stress from repeated sampling. Mice were restrained using a commercially available restraint, and a single droplet of blood obtained by puncturing the tail vein with a sterile lancet. Blood glucose concentrations were then assessed using a One Touch Basic glucometer (Lifescan, Milpitas, CA).

### Real Time quantitative RT-PCR (Q-PCR)

RNA was extracted from tissue samples using TRIZOL Reagent (Life Technologies) and reverse transcription was performed on 2 μg of RNA using a High-Capacity RNA-to-cDNA Kit (Life Technologies).Q-PCR was performed using the CFX96 Touch Real-Time PCR Detection System (Bio-Rad Laboratories, Hercules, CA, USA) using iQ SYBR Green Supermix (Bio-Rad Laboratories). The efficiency and specificity of each primer was assessed by generating standard curves to ensure amplification efficiency >90% and performing melt curve analysis to verify the production of a single gene product. Primers used are listed in Table 1 and the PCR program utilized was as follows: 10 min at 95°C, followed by 40 cycles of denaturation at 95°C for 15 seconds and annealing-elongation at 60°C for 1 minute. The acquisition of fluorescence data was performed at the end of the elongation step using CFX manager software V 2.1 (Bio-Rad Laboratories). Expression levels of each transcript were normalized by comparison with the amount of *18S* rRNA.

**Table 1:** PCR primers and sequences.

Gene	Accession number	Primer sequence 5’ to 3’		Amplicon size [bp]
*Per2*	NM_011066	Forward	GAAAGCTGTCACCACCATAGAA	186
		Reverse	AACTCGCACTTCCTTTTCAGG	
*Bmal1*	NM_007489	Forward	AACCTTCCCGCAGCTAACAG	79
		Reverse	AGTCCTCTTTGGGCCACCTT	
*Dbp*	NM_016974	Forward	CCTGAGGAACAGAAGGATGA	81
		Reverse	ATCTGGTTCTCCTTGAGTCTTCTTG	
*Reverb α*	NM_145434	Forward	TGCAGGCTGATTCTTCACAC	140
		Reverse	CAATCAGGATTCCAGGCACT	
*18S*	K01365.1	ForwardReverse	CTCTGTTCCGCCTAGTCCTG GGCCGTGCGTACTTAGACAT	123

### Data analysis

Results are presented as mean ± standard error of the mean (SEM). COSINOR analysis was done using the nonlinear regression model within SigmaPlot V 10.0 (Systat Software, San Jose, CA, USA). This program was used to assess rhythmicity of gene expression and to fit a cosine curve to the gene expression data. The model can be written according to the equation: f(x) = A+B cos [2 π(x) C) / 24] with the f(x) indicating relative expression levels of target genes, x indicating the time of sampling (h), A indicating the mean value of the cosine curve (mesor; midline estimating statistic of rhythm), B indicating the amplitude of the curve (half of the sinusoid) and C indicating the acrophase (h). Transcript levels were calculated relative to the average expression of each dataset throughout 24 hrs to plot temporal expression. One-Way ANOVA was used to compare the amplitude of clock gene expression between genotypes. Where the analysis of variance indicated a significant effect of genotype, a Holm-Sidak post-hoc test was performed. The level of significance for all tests was set at *p*<0.05.

## Results

### Effect of melatonin receptor deletion on daily rhythms in blood glucose

**Fig 1:**
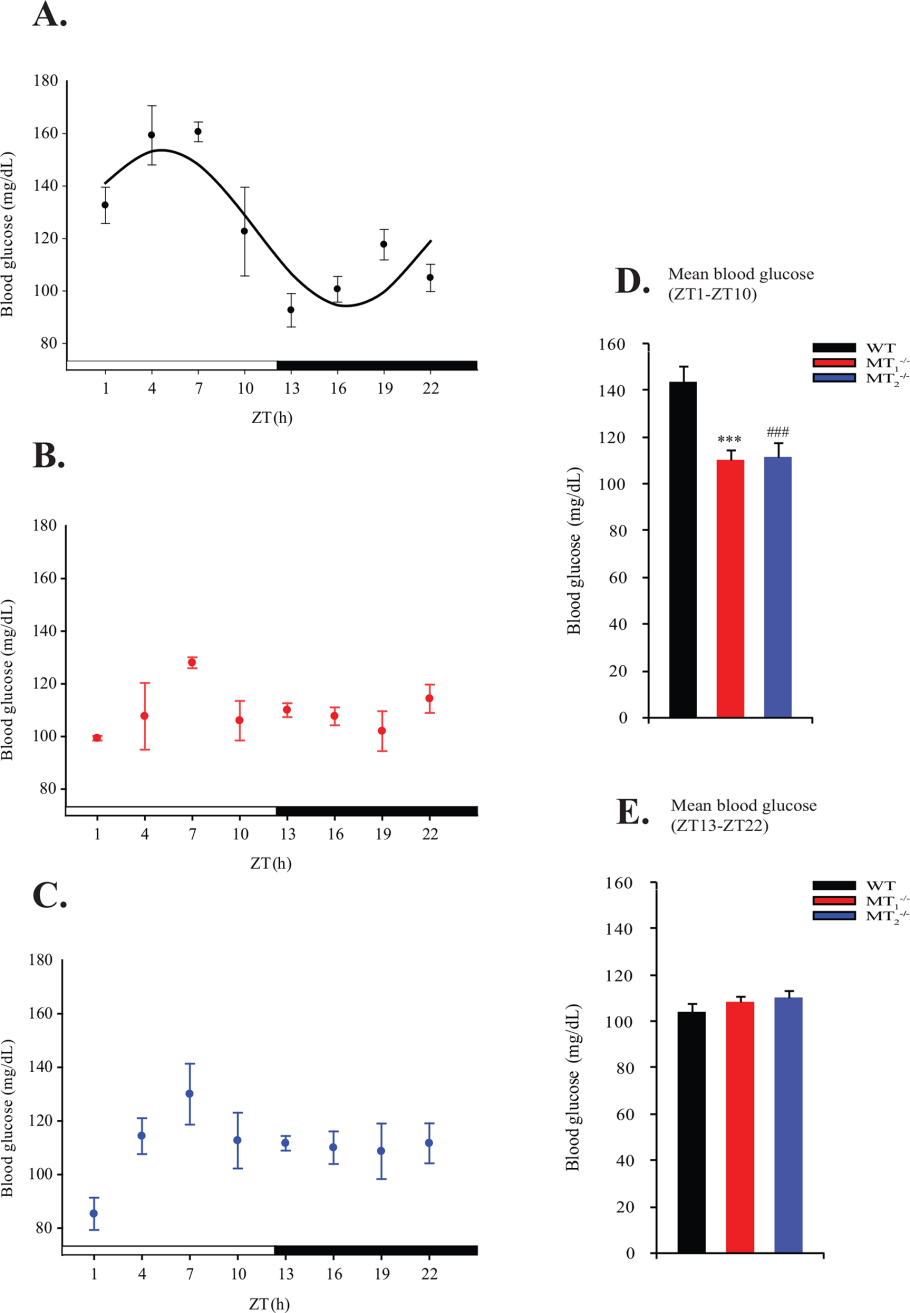
Loss of melatonin receptors disrupts daily rhythms in blood glucose. Blood glucose levels every 3 hours across the light/dark cycle in WT (A), MT_1_^-/-^ (B) and MT_2_^-/-^ (C) mice fed ad libitum are denoted within each respective plot. Results are expressed as mean ± SEM in which the black solid line in (A) represents the periodic sinusoidal function of the blood glucose rhythm as determined by COSINOR analysis (p<0.05, (N = 3–4 for each time point and genotype)). The light and dark bars below each plot (A-C) denote the light and dark phase respectively. Mean blood glucose levels for values collected during the light phase (ZT1-ZT10) and the dark phase (ZT13-22) are presented graphically in (D) and (E). ***p<0.001, One-Way ANOVA (post hoc: Holm-Sidak) WT vs. MT_1_^-/-^; ^###^p<0.001, One-Way ANOVA (post hoc: Holm-Sidak) WT vs. MT_2_^-/-^.

We first examined the effect of melatonin receptor deletion on the daily rhythm in blood glucose levels. As shown in Fig 1, Blood glucose concentrations in WT mice showed significant fluctuations over the light: dark cycle (One-Way ANOVA (post hoc: Holm-Sidak) p<0.001, Table 2: COSINOR, p<0.0001), with an amplitude (fold above trough) of 29.8 mg/dL and an estimated peak occurring at ~ ZT 5. Surprisingly, this rhythm was abolished in both MT_1_^-/-^ and MT_2_^-/-^ mice (One-Way ANOVA and COSINOR analysis produced p>0.05 for MT1 and MT_2_^-/-^ mice; Table 2). Interestingly, the loss of blood glucose rhythm in melatonin receptor knock-out mice coincided with differences in daytime glucose levels rather than nocturnal levels. Statistical analysis of glucose concentrations indicated that both MT_1_^-/-^ and MT_2_^-/-^ displayed reduced diurnal levels compared to WT mice (One-Way ANOVA (post hoc: Holm-Sidak); Fig 1 (D)).

**Table 2:** Blood glucose rhythms in WT, MT1^-/-^ & MT2^-/-^ mice.

Cosinor Parameter	WT		MT_1_^-/-^		MT_2_^-/-^	
**Mesor**	123.9±3.7	*p-value*	—-	*p-value*	—-	*p-value*
**Amplitude**	29.8±5.2	<0.0001	—-	0.38	—-	0.20
**Acrophase(h)**	4.6±0.7		—-		—-	

### Daily rhythms in clock and clock-controlled gene expression in skeletal muscle of WT, MT_1_^-/-^ and MT_2_^-/-^ mice

**Fig 2:**
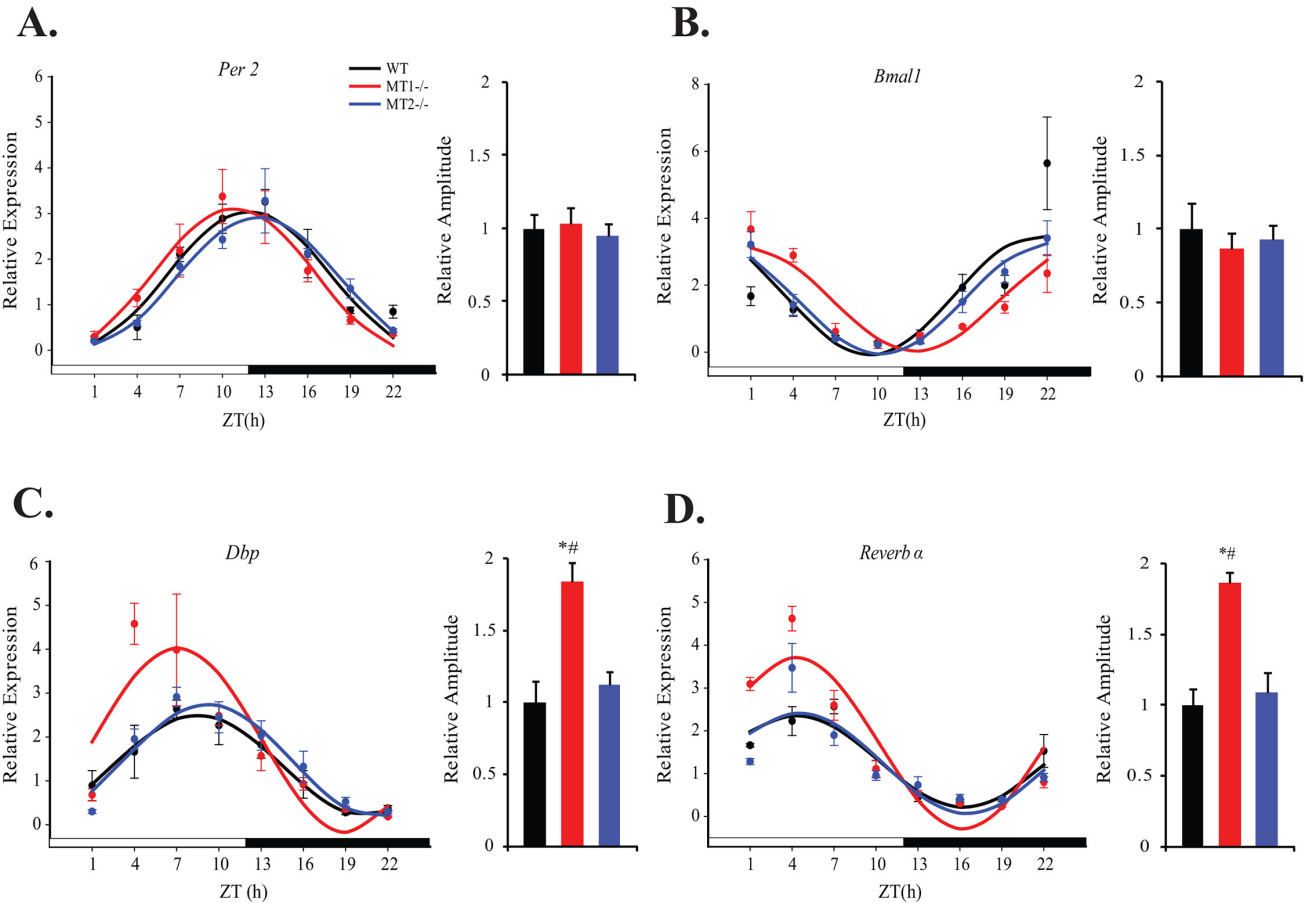
Expression levels of clock and clock controlled genes within skeletal muscle of WT, MT1^-/-^, and MT2^-/-^ mice. For each respective plot: the black circle (WT), red circle (MT_1_^-/-^) and blue circle (MT_2_^-/-^) indicate the expression levels of target genes calculated relative to the average expression of each dataset throughout the 24hrs. Results are represented as mean ± SEM. The black (WT), red (MT_1_^-/-^) and blue (MT_2_^-/-^) lines represent the periodic sinusoidal functions determined by COSINOR analysis (p<0.05, (N = 4–6 for each time point and genotype).The bar graphs corresponding to each plot depict the amplitude of each oscillating transcript as calculated by COSINOR analysis and normalized to the value of the WT amplitude. ***p<0.001, One-Way ANOVA (post hoc: Holm-Sidak) WT vs. MT_1_^-/-^; ^###^p<0.001, One-Way ANOVA (post hoc: Holm-Sidak) MT1^-/-^ vs. MT_2_^-/-^.

Recently, a vast array of metabolic phenotypes arising from tissue specific clock gene mutant mice [30–37] has shed light on the importance of peripheral tissue clocks in the regulation of glucose homeostasis. To begin investigating the influence of melatonin receptor deletion on rhythmic clock gene expression within insulin sensitive tissues, relative mRNA levels were analyzed within skeletal muscle of WT, MT_1_^-/-^ and MT_2_^-/-^ mice (Fig 2 and Table 3). In both WT and melatonin receptor knock-out mice, clock genes (*Per2*, *Bmal1 and Reverb α)* and the clock controlled gene *Dbp* were rhythmically expressed (Table 3, COSINOR, p<0.0001). *Bmal1* and *Per2* demonstrated a typical antiphasic expression pattern; with an expression peak or trough for *Bmal1* occurring at ZT 22 or ZT 11 and *Per2* occurring during the day at ZT11 or ZT 22, respectively. As expected, *Dbp* also exhibited a maximum peak during the mid-light phase. The most notable effects of melatonin receptor deletion on the skeletal muscle clock were on the amplitude of *Dbp* and *Reverb* α. MT_1_^-/-^ mice exhibited a ~2-fold increase in amplitude for both transcripts, compared to WT and MT_2_^-/-^ mice (One-Way ANOVA (post-hoc: Holm-Sidak), p<0.001). No differences in the peak phase of *Per2*, *Dbp* and *Reverb α* were observed among the three genotypes, however with respect to *Bmal1*, a very subtle phase advance was present in MT_1_^-/-^ mice with respect to WT and MT_2_^-/-^ mice (One- Way ANOVA (post-hoc: Holm-Sidak), p<0.001; Table 3). Taken together this data suggests that removal of melatonin receptors confers rather small effects on the rhythmic expression pattern of clock genes within skeletal muscle.

**Table 3:** COSINOR analysis of clock controlled genes within skeletal muscle of WT, MT1^-/-^ amd MT2^-/-^ mice.

Gene	Cosinor Parameter		WT				MT_1_^-/-^				MT_2_^-/-^		
			Output		Regression p-value		Output		Regression p-value		Output		Regression p-value
***Per2***	Mesor		1.58±0.10		<0.0001		1.58±0.12		<0.0001		1.52±0.08		<0.0001
	Amplitude		1.47±0.14				1.52±0.17				1.40±0.12		
	Acrophase(h)		11.87±0.37				10.83±0.43				12.51±0.31		
***Bmal1***	Mesor		1.70±0.22		<0.0001		1.57±0.12		<0.0001		1.60±0.11		<0.0001
	Amplitude		1.79±0.31				1.55±0.17				1.66±0.16		
	Acrophase(h)		21.43±0.66				0.70±0.44				22.20±0.38		
***Dbp***	Mesor		1.35±0.12		<0.0001		1.93±0.19		<0.0001		1.46±0.09		<0.0001
	Amplitude		1.14±0.17				2.10±0.28				1.28±0.12		
	Acrophase(h)		8.50±0.58				7.07±0.49				9.21±0.38		
***Reverbα***	Mesor		1.28±0.08		<0.0001		1.71±0.11		<0.0001		1.24±0.11		<0.0001
	Amplitude		1.07±0.12				2.00±0.15				1.17±0.16		
	Acrophase(h)		4.28±0.41				4.23±0.29				4.52±0.50		

### Daily rhythms in clock and clock-controlled gene expression within liver and adipose tissue of WT, MT_1_^-/-^ and MT_2_^-/-^ mice

**Fig 3:**
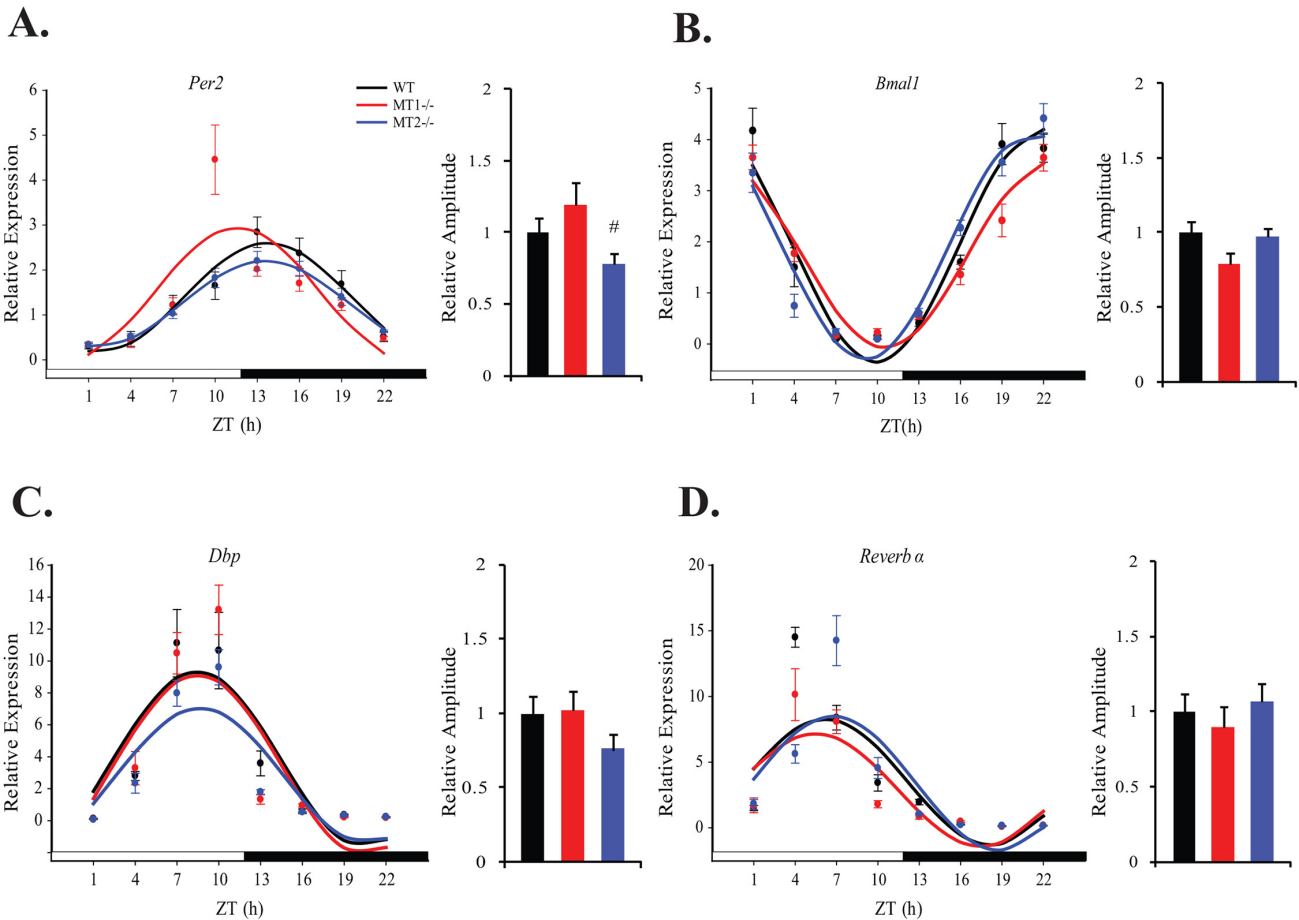
Expression levels of clock and clock controlled genes within liver of WT, MT1^-/-^, and MT2^-/-^ mice. For each respective plot: the black circle (WT), red circle (MT_1_^-/-^) and blue circle (MT_2_^-/-^) indicate the expression levels of target genes calculated relative to the average expression of each dataset throughout the 24hrs. Results are represented as mean ± SEM. The black (WT), red (MT_1_^-/-^) and blue (MT_2_^-/-^) lines represent the periodic sinusoidal functions determined by COSINOR analysis (p<0.05, (N = 4–6 for each time point and genotype). The bar graphs corresponding to each plot depict the amplitude of each oscillating transcript as calculated by COSINOR analysis and normalized to the value of the WT amplitude. ^#^p<0.05, One-Way ANOVA (post-hoc: Holm-Sidak) MT1^-/-^ vs. MT_2_^-/-^.

**Fig 4:**
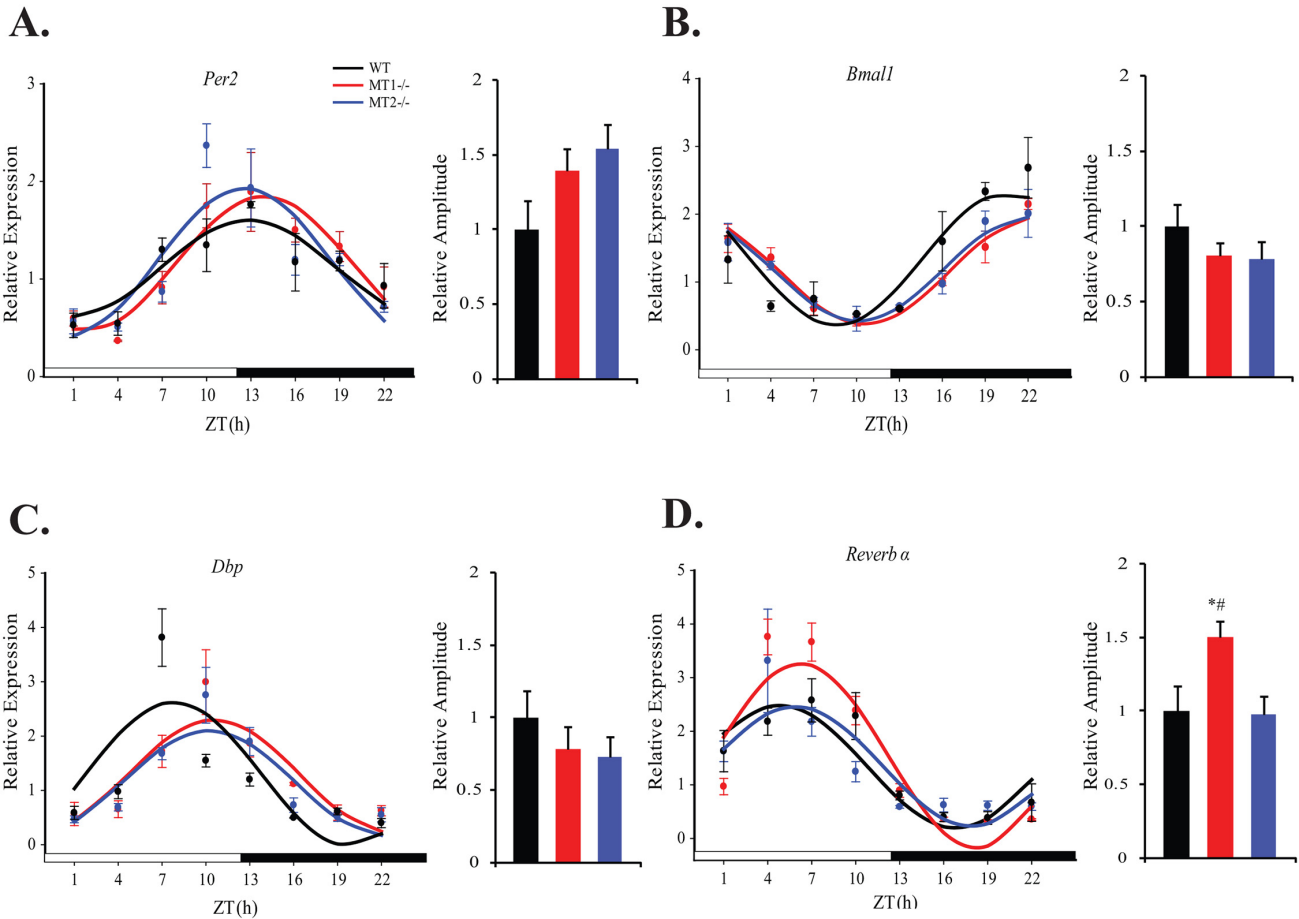
Expression levels of clock and clock controlled genes within adipose tissue of WT, MT1^-/-^, and MT2^-/-^ mice. Daily gene expression levels of *Per2* (A), *Bmal1* (B), *Dbp* (C) and *Reverb α* (D) are depicted above. For each respective plot: the black circle (WT), red circle (MT_1_^-/-^) and blue circle (MT_2_^-/-^) indicate the expression levels of target genes calculated relative to the average expression of each dataset throughout the 24hrs. Results are represented as mean± SEM. The black (WT), Red (MT_1_^-/-^) and blue (MT_2_^-/-^) lines represent the periodic sinusoidal functions determined by COSINOR analysis (p<0.05, (N = 4–6 for each time point and genotype). The bar graphs corresponding to each plot depict the amplitude of each oscillating transcript as calculated by COSINOR analysis and normalized to the value of the WT amplitude. *p<0.05, One-Way ANOVA (post-hoc: Holm-Sidak) WT vs. MT_1_^-/-^; ^#^p<0.05, One-Way ANOVA (post-hoc: Holm-Sidak) MT1^-/-^ vs. MT_2_^-/-^.

Regulation of blood glucose levels occurs as a concerted effort between skeletal muscle, liver, and adipose tissue. Therefore in addition to skeletal muscle, we examined clock gene expression within liver and adipose tissue of melatonin receptor knock-out mice. For both liver and adipose tissue, clock and clock controlled genes were rhythmic in all three genotypes (Tables 4 & 5, COSINOR, p<0.0001). In WT mice, *Per2* peaked at ~ ZT14, *Bmal1* peaked at ~ ZT21, *Dbp* ~ ZT 8 and *Reverb* α ~ZT 5 (Figs 3 & 4, Tables 4 & 5). Removal of either MT_1_ or MT_2_ receptors produced subtle effects on phase and amplitude of these rhythms in both tissues. In the liver, removal of MT_2_ resulted in a significant reduction in the amplitude of *Per2* with respect to MT_1_^-/-^ mice (One-Way ANOVA (post-hoc: Holm-Sidak), p<0.05)—this effect was not present in adipose tissue or skeletal muscle. Similar to the effect observed in skeletal muscle, MT_1_-deficiency resulted in a significant increase in the amplitude of *Reverb α* in adipose tissue, with respect to WT and MT_2_^-/-^ mice (One-Way ANOVA (post-hoc: Holm-Sidak), p<0.05) -this effect was not present in the liver. Taken together, these results demonstrate that the removal of melatonin receptors induces rather small, tissue-specific effects on the rhythmic expression pattern of clock genes, suggesting that function of the molecular circadian clockwork is not drastically affected by the loss of melatonin receptors. Thus, the effect of melatonin receptor deficiency on daily glucose rhythms appears to be independent of molecular clock function.

**Table 4:** COSINOR analysis of clock genes and clock controlled genes within the liver of WT, MT1^-/-^ and MT2^-/-^ mice.

Gene	Cosinor Parameter		WT				MT_1_^-/-^				MT_2_^-/-^		
			Output		Regression p-value		Output		Regression p-value		Output		Regression p-value
***Per2***	Mesor		1.39±0.09		<0.0001		1.48±0.16		<0.0001		1.25±0.05		<0.0001
	Amplitude		1.22±0.12				1.46±0.22				0.95±0.07		
	Acrophase(h)		13.75.±0.38				11.59±0.59				13.58±0.29		
***Bmal1***	Mesor		1.92±0.12		<0.0001		1.74±0.08		<0.0001		1.91±0.09		<0.0001
	Amplitude		2.28±0.17				1.81±0.12				2.21±0.13		
	Acrophase(h)		21.90±0.29				22.54±0.24				21.15±0.22		
***Dbp***	Mesor		3.86±0.48		<0.0001		3.51±0.49		<0.0001		2.83±0.33		<0.0001
	Amplitude		5.49±0.66				5.62±0.71				4.22±0.46		
	Acrophase(h)		8.44±0.47				8.48±0.45				8.65±0.41		
***Reverbα***	Mesor		3.48±0.42		<0.0001		2.87±0.42		<0.0001		3.40±0.49		<0.0001
	Amplitude		4.79±0.59				4.29±0.60				5.10±0.63		
	Acrophase(h)		6.18±0.47				5.48±0.53				6.77±0.46		

**Table 5:** COSINOR analysis of clock genes and clock controlled genes within adipose tissue of WT, MT1^-/-^ and MT2^-/-^ mice.

Gene	Cosinor Parameter		WT				MT_1_^-/-^				MT_2_^-/-^		
			Output		Regression p-value		Output		Regression p-value		Output		Regression p-value
***Per2***	Mesor		1.11±0.06		<0.0001		1.16±0.07		<0.0001		1.17±0.09		<0.0001
	Amplitude		0.49±0.10				0.69±0.10				0.76±0.13		
	Acrophase(h)		12.84±0.68				13.89±0.56				12.56±0.64		
***Bmal1***	Mesor		1.34±0.10		<0.0001		1.16±0.05		<0.0001		1.19±0.06		<0.0001
	Amplitude		0.98±0.14				0.79±0.07				0.77±0.09		
	Acrophase(h)		20.58±0.58				22.56±0.33				22.11±0.45		
***Dbp***	Mesor		1.30±0.18		<0.0001		1.27±0.12		<0.0001		1.14±0.09		<0.0001
	Amplitude		1.32±0.24				1.03±0.16				0.96±0.14		
	Acrophase(h)		7.79±0.76				10.55±0.60				10.20±0.52		
***Reverbα***	Mesor		1.34±0.14		<0.0001		1.54±0.14		<0.0001		1.35±0.10		<0.0001
	Amplitude		1.14±0.19				1.72±0.19				1.12±0.14		
	Acrophase(h)		4.81±0.67				6.23±0.49				5.88±0.57		

## Discussion

Within our bodies a single day can be partitioned into two separate metabolic phases- one characterized by activity and feeding, and the other by rest and fasting. In this regard, it is critical that metabolic timing cues be properly distributed to temporally separate distinct metabolic functions. The temporal coordination of glucose metabolism is critical for the maintenance of glucose homeostasis and energy balance [38]. Disturbances in glucose availability and insulin action have been linked to a variety of metabolic disorders such as obesity, metabolic syndrome, and type 2 diabetes. Notably, one of the most pronounced rhythmic aspects of physiology within both humans and rodents is the daily regulation of blood glucose levels across the 24hr day [17–21].

In the present study we report that melatonin signaling regulates the daily rhythm in blood glucose levels, independent of clock gene expression within insulin sensitive tissues. Melatonin proficient mice containing both melatonin receptors (MT_1_ & MT_2_) exhibited a clear rhythm in blood glucose levels marked by a diurnal peak occurring prior to the onset of activity (Fig 1). This observation is in agreement with previous studies in both rodents and humans in which glucose concentrations rise prior to onset of activity [19, 39–40]. Albeit some discrepancies in the precise timing of this rise, with studies in rats and humans depicting a more defined ‘awakening’ rise in blood glucose levels. These inherent fluctuations in glucose concentrations are dependent on the SCN, occur independently of feeding rhythms, and can be attributed to temporal variations in both hepatic glucose production and glucose tolerance [41]. In this way, the SCN is thought to play a role in preparing an individual for the upcoming activity period by increasing plasma glucose concentrations and facilitating tissue glucose uptake.

Interestingly, within both MT_1_^-/-^ and MT_2_^-/-^ mice the daily rhythm in blood glucose levels was abolished (Fig 1), thereby suggesting a role for melatonin receptors in the regulation of this rhythm. Our results differ slightly from those of a previous report by Muhlbauer et al. [28] demonstrating that MT_1_^-/-^ have increased mean blood glucose levels over a 24 hr. period. The discrepancy between our findings and theirs could be due to the age of the mice and the precise bleeding protocol used in each study. In our study we utilized mice 3–4 months of age while the study by Muhlbauer et al. assessed mice at 1.5 months of age; additionally it is not clear whether this study used a repeated bleeding protocol or single bleed protocol to assess the blood glucose rhythm. As the loss of blood glucose rhythm was observed when either MT_1_ or MT_2_ was knocked out, it raises the interesting possibility that the maintenance of this rhythm could involve signaling through the formation of MT_1_/MT_2_ heteromers [42]. Recently, a functional role for these heteromers was demonstrated in the retina where deletion of either MT_1_ or MT_2_ abolished the daily rhythm in electroretinogram (ERG) responses [43].

Glucose homeostasis is known to involve the concerted effort of the hypothalamic clock in the SCN as well as peripheral clocks within insulin sensitive tissues such as skeletal muscle, the liver, and adipose tissue [44–47]. To date, a number of metabolic phenotypes have been produced by tissue specific clock gene knock-out mice and serve to highlight the diverse role of the circadian clock in regulating whole body glucose metabolism. Muscle specific *Bmal1*^-/-^ mice are insulin resistant and have reduced protein levels of GLUT4[30], while liver specific deletion of *Bmal1* leads to a stark increase in glucose tolerance[31]. Interestingly, adipose specific *Bmal1*^-/-^ mice become obese [34], whereas deletion within the pancreas results in defective β-cell function [32,33,35].

Given the loss of the blood glucose rhythm in MT_1_^-/-^ and MT_2_^-/-^ mice, it was rather surprising that we observed only marginal effects on rhythmic clock gene expression within insulin sensitive tissues of MT_1_^-/-^ and MT_2_^-/-^ mice. Indeed, previous studies have established a role for melatonin signaling in the regulation of clock gene expression [24–26]. In our analysis, the most evident effects observed by melatonin receptor deletion, were tissue specific increases in the amplitude of clock controlled transcripts within MT_1_^-/-^ mice. Within skeletal muscle, removal of MT_1_ resulted in a nearly 2 fold increase in the amplitudes of *Reverb* α and *Dbp* (Fig 2; Table 3); and within adipose tissue a 1.5 fold increase in the amplitude of *Reverb* α (Fig 4; Table 5). Our findings within adipose tissue, are consistent with a recent study in which pinealectomy increased the amplitude of *Reverb* α at its peak [48]. As we observed no coincident changes on *Reverb* α amplitude in MT_2_^-/-^ mice, this could suggest a receptor specific role for MT_1_ signaling in dampening the daily amplitude of Reverb α within adipose tissue and skeletal muscle. Interestingly, strong amplitude increases in *Dbp* and *Reverb* α have been reported within the pancreas of MT_2_^-/-^ mice, with no effect in MT_1_^-/-^ mice [28]- further substantiating tissue specific roles on the modulation of clock gene expression by MT_1_ and MT_2_. In all tissues observed, increases in the amplitude of Reverb α were not accompanied by a coincident decreases in the expression level of *Bmal1*, implying that additional modulatory effects may be in place to regulate the amplitude of *Bmal1* [49].

In the present study we demonstrate that daily variations in blood glucose levels do not directly depend on rhythmic cycling of clock genes within insulin sensitive tissues, as evidenced by the fact that the disruption of this rhythm in melatonin receptor knock-out mice occurred independently of clock disruption. The identification of numerous tissue specific circadian transcripts within peripheral tissues highlights that circadian regulation extends far beyond core clock components [50,51]. Furthermore it has now become increasingly evident that the timing of the biological clock is subject to a plethora of post-transcriptional and post-translational regulatory mechanisms[52]. Therefore we cannot rule out the possibility that melatonin signaling could indirectly affect blood glucose rhythms by altering the functioning of the clock at the post transcriptional or post-translational level. Along these lines recent studies have begun to suggest a potential role for melatonin in proteosomal degradation [53,54].

It has been shown that the increase in blood glucose concentrations prior to the onset of activity occurs as a result of increased glucose production by the liver rather than decreased glucose utilization by insulin sensitive tissues[41]. Therefore the clear daytime blunting in the amplitude of the blood glucose rhythm within melatonin receptor knock-out mice likely reflects alterations in hepatic glucose production. Indeed previous studies have suggested that melatonin is capable of activating a hypothalamic-liver communication which may contribute to circadian adjustments of gluconeogenesis by suppressing gluconeogenesis [55,56]. Furthermore pinealectomized rats display glucose intolerance and a desynchronized pattern of gluconeogenesis, marked by increased nighttime glucose levels[27]. All these notions are consistent with a role for nocturnal secretion of melatonin lowering night-time glucose concentrations. Here we demonstrate that global disruption of melatonin receptors does not affect nocturnal glucose levels, and in fact appears to have a more pronounced effect during the day. This could suggest a more indirect effect of melatonin on the temporal regulation of gluconeogenesis. Future studies would need to be done to further elucidate indirect and direct effects of melatonin receptor signaling on gluconeogenesis.

The loss in blood glucose rhythms observed in melatonin receptor knock-out mice does not appear to have resulted from differences in the food intake rhythm (personal communication with Charlotte von Gall). These results are consistent with pinealectomy studies demonstrating that removal of melatonin has no apparent effects on the rhythm of food intake[57]. It is possible that the loss of the blood glucose peak could result from increased insulin concentrations after melatonin receptor deletion. Although there have been reports of a circadian rhythm in insulin secretion[17], studies demonstrating that fasted rats do not show an insulin rhythm[58], suggest that the food intake rhythm drives the rhythm in insulin secretion. Therefore it is unlikely that major differences in the insulin secretory response occur independently of food intake within melatonin receptor knock-out mice.

Taken together our data demonstrates that melatonin receptors are critical for the temporal regulation of glucose homeostasis. Given that no coincident disturbances were found in the rhythmic expression of clock genes within insulin sensitive tissues, it could suggest that this regulation occurs at the level of the central nervous system-possibly the SCN- by a mechanism which involves coupling of nutrient sensing by the brain to glucose production by the liver. Alternatively, an additional explanation could be that melatonin signaling acts directly on the expression/activity of genes involved in glucose metabolism. Peripheral regulation alone, for example melatonin binding to receptors within the pancreas, skeletal muscle or liver is unlikely as the effects of melatonin receptor deletion on blood glucose levels are not restricted to the dark phase (time window when melatonin is secreted). Future studies will be needed to further elucidate the role of melatonin receptor signaling in the regulation of the daily regulation of blood glucose levels.
